# Naturalist's Monthly Report

**Published:** 1811-09

**Authors:** 


### NATURALIST'S MONTHLY REPORT.

### JULY.

#### FRUITING MONTH.

Now comes July, and with his fervid noon
Unnerves the hand of toil. The mower sleeps—
The sun-burnt maid rakes feebly—the hot swain
Pitches his load reluctant—the faint steer,
Lashing his sides, draws heavily along
The slow encumber'd wain.

The weather has, on the whole, been more than usually cold during the present month, owing in a great measure to the general prevalence of northerly winds. On the 1st the wind was north-east; on the 2d variable; on the 3d and 4th north-east; on the 5th, 6th and 7th, variable; on the 8th north-east; on the 9th north-west; on the 10th variable; on the 11th and 12th north-west; on the 13th, 14th and 15th, westerly; on the 16th, 17th, 18th and 19th, south-west; on the 20th and 21st northerly; on the 22d, 23d, and 24th, westerly; on the 25th north-west; on the 26th north-east; on the 27th and 28th variable; on the 29th south-east; on the 30th variable, and on the 31st easterly.

There were strong gales on the 3d, 14th, and 22d, and fresh gales on the 4th, 12th, 13th, 16th, 17th, 18th, 19th, 23d and 25th. We had rain, more or less, on the 2d, 3d, 10th, 15th, (St. Swithin) 18th, 19th, 20th, 21st and 22d. On the 2d the showers were sxcessively heavy heavy, and accompanied with thunder; this, however, was the only thunder-storm we had during the whole month.

July 3d. The young swallows begin to leave their nests. The bloom of the lime tree drops off.

July 4th. Apricots are ripe. In this part of the country fruit of almost all kinds is now extremely scarce, owing to the frosty nights which occurred during the time in which the trees were in bloom. Gooseberries and currants are nearly the only kinds which do not appear to have suffered injury. The apples in several parts of Hampshire are fewer in quantity than have been known for many years past.

July 8. A lamprey (*petromyzon marinus* of Linnæus) was this day brought to me. These fish, although in some places held in the highest esteem for the tables of the opulent, are here entirely neglected. No one, in this neighbourhood at least, appears inclined to risk the fate of our King Henry the first, who died in consequence of a surfeit by eating too voraciously of them. Lampreys are inhabitants of the sea, but come up the rivers, in the spring of the year, for the purpose of depositing their spawn. It is about the months of June and July that in our rivers they are usually caught, but as they are in no request, the fishermen seldom expose them to sale.

July 11th. The mackarel fishers have been very unsuccessful, except during a few days at the commencement of the season.

July 14th. Bank martins (*hirundo riparia* of Linnæus) have left their nests and fly about.

Common dodder (*cuscuta Europæa*), bog pimpernel (*anagallis tenella*), marsh cinquefoil (*comarum palustre*), hare's foot trefoil (*trifolium arvense*), hoary cinquefoil (*potentilla argentea*), jointed rush (*juncus articulatus*), hard rush (*juncus inflexus*), wild teasel (*dipsacus fullonum*), bull-rush or reed-mace (*typha latifolia*), great bind-weed (*convolvulus sepium*), and yellow stone-crop (*sedum reflexum*), are now in flower.

July 15th. St. Swithin. The omen of rain for forty successive days, by rain having fallen on the commemoration day of the Winchester saint, has this year entirely failed.

July 20th. The rye is nearly ripe. The barley and wheat are now quite yellow; and the crops for the most part extremely abundant, notwithstanding the outcry respecting a blight, which has been with great industry spread abroad by a few of the farmers, for the purpose of enhancing the price of the grain now on hand.

July 23d. A salmon of considerable weight, which it is supposed had been struck by a porpoise, was caught by one of the bathing women within a few yards of the shore. It was still alive.

July 26th. Field peas are cut. Morella cherries are ripe.

July 27th. I this day heard, for the first time, the shrill, continued crinking of the large green locust (*gryllus verrucivorus* of Linnæus).

July 31st. Oats, rye, and wheat have been cut.

The swallow tribe appear to be congregating much earlier than usual. This, however, I presume, is entirely owing to the cold weather which has of late been so prevalent; and no doubt, when the weather again becomes seasonable, they will again disperse, until the regular period of their migration approaches.

*Hampshire.*

